# At What Age Does Menstration Cease?

**Published:** 1848-06

**Authors:** 


### KINGSTON ASSIZES.

*At what age does Menstruation cease? Singular Question of Identity.*—In this case, which was an action of ejectment, the identity of a female rested in some degree upon the period of life at which the menses ceased. The plaintiff, Clark, claimed an estate through his wife, to whom he was married in 1794. They had three children, but, in consequence of disagreement, separated in 1809. The wife, it was alleged, died in 1843, and the defendant, Tatom, took possession of some property belonging to her. The husband now sought to recover this property. One witness deposed that she knew Mrs. Clark, and that she was about seventy years old when she died. This would have made her age at marriage twenty-one. Another witness, who provided her funeral, put on her coffin-lid, by the direction of defendant, the age of fifty-five; and stated that he should have considered her to have been about that age, or not more than two or three years older. If this were true, the deceased Mrs. Clark must have been married when she was six years old! This witness afterwards said that there was another Mrs. Clark still living at Norword (the place where deceased resided), who appeared to be between fifty and sixty years old. This was an unfortunate admission, because, taking the age of the surviving Mrs. Clark at fifty-five, she must also have been married at the age of six years. The unlucky witness, therefore, proved that the plaintiff either married in 1794 a girl of six years of age, or that neither the surviving nor the deceased Mrs. Clark could have been his wife, when the evidence clearly showed that he must have been married to one or the other.

In this stage of confusion in which matters were left by the undertaker, medical evidence was called, in order to prove the non-identity of the deceased female with the lady whom the plaintiff married in 1794.

"Dr. Laurie, a physician who was in practice at Norwood in 1841, deposed that he had attended Mrs. Clark (the deceased); and at that time she appeared to be about fifty years old. The witness then detailed the circumstances under which he was called upon to attend the deceased, referring to a particular change in the female constitution which, he said, rarely occurred to them so late as the age of fifty, the general period being forty-four.

"Cross-examined: He judged of her age from the colour of her hair and other circumstances (?). He saw nothing to induce him to believe that she was more than fifty years old when he attended upon her.

"Dr. Alison, another physician, gave similar testimony."

If the evidence adduced by the plaintiff were correct, and the medical opinion in accordance with medical experience and doctrine, it followed that the woman, who was not more than fifty in 1841, must have been married to her husband, the plaintiff, when she was three years old! Thus the case was rendered a little more complex than it had been left by the undertaker.

It was well observed by the Lord Chief Justice that the case was full of difficulties. According to the testimony of a most respectable witness (Mrs. Hudson) the plaintiff's case was completely established, for she swore positively that the Mrs. Clark, who died at Norwood in 1843, was the wife of the plaintiff; but on the other hand the medical testimony, which appeared very clear and decisive, rendered it equally impossible that she could have been the same person. The jury found for the plaintiff.—*London Medical Times.*

